# Governing medical AI under the EU AI Act: an integrated compliance-by-design framework

**DOI:** 10.3389/fdgth.2026.1932256

**Published:** 2026-09-09

**Authors:** Qaiser Khan, Abdul Raffay Saeed

**Affiliations:** Empirical Software Engineering in Software, Systems and Services (M3S), Faculty of Information Technology and Electrical Engineering, University of Oulu, Oulu, Finland

**Keywords:** compliance-by-design, conformity assessment, data governance, EU AI Act, high-risk AI systems, medical AI, medical device regulation

## Abstract

Artificial intelligence (AI) is becoming an increasingly important component of healthcare, supporting diagnosis, clinical decision-making, medical imaging, triage, and patient-facing services. While these technologies offer significant opportunities to improve healthcare delivery, they also raise important questions about patient safety, fairness, privacy, transparency, cybersecurity, and accountability. This paper examines the regulatory framework governing medical AI in the European Union, focusing on the interaction between the Artificial Intelligence Act (AI Act), the Medical Device Regulation (MDR), the In Vitro Diagnostic Medical Device Regulation (IVDR), the General Data Protection Regulation (GDPR), and the European Health Data Space (EHDS). Drawing on doctrinal and conceptual regulatory analysis, it argues that many AI-enabled medical systems should be regulated through continuous lifecycle compliance rather than by relying primarily on a one-time assessment at market entry. The analysis identifies the training and validation dataset as a particularly important point of regulatory convergence, especially among the EHDS, GDPR, and AI Act data-governance requirements, and examines how responsibility is allocated across the general-purpose AI value chain under Article 25. Building on this analysis, the paper proposes a compliance-by-design framework that translates overlapping legal requirements into a set of auditable governance artefacts covering system classification, regulatory mapping, data governance, validation, human oversight, conformity assessment, post-market monitoring, and change control. The framework is illustrated through two contested use cases and complemented by recommendations tailored to the responsibilities of different stakeholder groups. The analysis incorporates Regulation (EU) 2026/1744 (Digital Omnibus on AI), while recognising that supporting guidance, harmonised standards, and implementation of the EHDS continue to evolve and should be checked when the framework is applied. The framework is conceptually derived and illustrated through constructed use cases rather than empirically validated; future work should assess its usability, consistency, and discriminating power through structured expert assessment and Delphi-based consensus methods.

## 1 Introduction

Artificial intelligence (AI) is rapidly becoming part of routine healthcare practice, embedded in software as a medical device, diagnostic imaging, pathology, clinical decision support, remote monitoring, triage, and patient-facing applications. These technologies offer significant opportunities to improve diagnostic accuracy, personalise treatment, and enhance healthcare delivery, while raising important concerns about patient safety, fairness, transparency, accountability, cybersecurity, and privacy (1–3).

The EU Artificial Intelligence Act (AI Act), Regulation (EU) 2024/1689, establishes the world’s first comprehensive legal framework for AI systems (4). Unlike earlier approaches that relied on ethical principles and voluntary guidance, the AI Act introduces legally binding obligations based on a risk-based model. In healthcare, these obligations coexist with the MDR, IVDR, GDPR, and EHDS, which the AI Act supplements rather than replaces (5–9).

Existing research addresses medical AI governance from several complementary perspectives, including legal analyses of individual or interacting regulatory regimes, healthcare-specific governance frameworks, Responsible AI approaches, and emerging certification-oriented engineering methods. These contributions provide important elements of lifecycle governance, but they address different subsets of the regulatory and implementation problem. The gap addressed in this paper is therefore narrower: how to coordinate the AI Act, MDR, IVDR, GDPR, and EHDS within a single lifecycle architecture that links regulatory obligations to inspectable governance artefacts.

This paper’s main contribution is a proposed unified compliance-by-design pipeline that operationalises legal checks across the AI Act, MDR, IVDR, GDPR, and EHDS throughout the lifecycle of high-risk medical AI. It makes three related contributions. First, it integrates the five regimes, including responsibility allocation along the general-purpose AI value chain under Article 25. Second, it treats compliance as a continuous process from design through deployment, monitoring, and modification. Third, it links the overlapping legal obligations at each lifecycle stage to auditable governance artefacts and clearly allocated responsibilities that can be inspected within existing assurance and conformity-assessment processes. The term “compliance by design” deliberately extends into the AI Act, setting the data-protection-by-design logic established in Article 25 GDPR (7), and is theoretically grounded in the regulation-by-design and meta-regulation literature discussed in Section 7 (10, 11). The framework is proposed as a structured governance model for practical implementation rather than an empirically validated compliance methodology; the worked examples illustrate its application, while empirical evaluation remains a separate next step.

Throughout the paper, *providers* refers to the natural or legal persons who develop or place AI systems on the market under their own name, as defined in Article 3(3) of the AI Act; *deployers* refers to those who use AI systems under their authority in a professional context, as defined in Article 3(4). The remainder of the paper is organised as follows. Section 2 describes the methodology. Section 3 introduces the principal categories of medical AI. Section 4 examines the AI Act’s risk-based architecture and its interaction with the five legal regimes. Section 5 analyses the substantive obligations applicable to high-risk medical AI. Section 6 discusses two regulatory boundary questions. Section 7 positions the proposed approach within the existing governance literature before Section 8 introduces the compliance-by-design framework. Section 9 demonstrates the framework through two worked use cases, followed by actor-specific recommendations in Section 10. Finally, Sections 11, 12, 13, and 14 discuss implementation challenges, the legislative timeline, study limitations, and conclusions.

## 2 Methods: conceptual and regulatory analysis

This is a doctrinal and conceptual paper rather than an empirical one. The objective is to analyse the interaction between the principal European legal frameworks governing medical AI and to translate their overlapping regulatory requirements into an operational governance framework. Methodologically, the analysis consists of a combination of doctrinal synthesis (deducing the obligations created under each of the instruments through their own primary sources), purposive interpretation (interpretation of contentious provisions in the context of the goal of protection, in keeping with the approach adopted by the Court of Justice of the European Union), and comparative regulatory analysis (utilising the US change control system as the external comparator). The analysis takes place through reasoning from primary sources of law, regulatory documents, and interdisciplinary literature to create a structured lifecycle model for compliance.

The legal analysis focuses on the AI Act, MDR, IVDR, GDPR, and EHDS. Each instrument was examined to identify its scope, triggering conditions, substantive obligations, and relationship with the other applicable regimes. Where instruments imposed overlapping or competing requirements, the analysis generally treated the more specific and more protective obligation as controlling, resolving apparent tensions by reference to the system’s intended purpose and to the relationship between the AI Act as horizontal law and the MDR/IVDR as *lex specialis*; residual conflicts are flagged as open boundary questions in Section 6. The analysis was further informed by *SNITEM and Philips France* (C-329/16) and by guidance from the European Commission, MDCG, IMDRF, WHO, and FDA. The analysis incorporates regulatory developments available at the time of revision, including Regulation (EU) 2026/1744 (Digital Omnibus on AI). Throughout the analysis, binding requirements are distinguished from regulatory guidance, the authors’ interpretation of unsettled provisions, and implementation recommendations. Mandatory language is reserved for requirements derived from applicable legislation, while interpretive positions and recommended practices are identified as such in the text and worked examples.

Academic literature was used to contextualise the legal analysis and characterise emerging implementation challenges rather than to provide an exhaustive review. Sources were identified through Scopus, Web of Science, PubMed, and Google Scholar and selected on the basis of relevance, scholarly standing, and regulatory authority. Priority was given to peer-reviewed articles, official legislation, regulatory guidance, international standards, and widely recognised reporting frameworks, including CONSORT-AI, DECIDE-AI, FUTURE-AI, CLAIM, and QUEST. No formal systematic review protocol was applied because the objective was conceptual framework development. For the comparative analysis in Section 7, frameworks were selected purposively as representative examples of distinct approaches relevant to the study’s contribution, including principle-based governance, international risk and management standards, healthcare-specific implementation frameworks, adaptive change-control approaches, EU medical-AI legal analysis, and certification-orientated Responsible AI engineering. The comparison is therefore illustrative rather than exhaustive and is used to position the proposed framework relative to these complementary approaches rather than to establish the absence of all comparable work. The findings were synthesised into a compliance-by-design framework organised into a sequence of lifecycle governance stages, each associated with a defined artefact. Empirical validation falls outside the scope of this study; limitations and priorities for future evaluation are discussed in Section 13.

## 3 Medical AI: categories and regulatory relevance

From a regulatory perspective, the defining consideration for any medical AI system is not the underlying technique but its intended medical purpose, clinical role, deployment context, and potential impact on patient safety and fundamental rights (1, 2, 12). Many medical AI systems are deployed as Software as a Medical Device (SaMD), performing one or more medical purposes without forming part of a hardware device (12), or as AI components embedded within medical devices. Such systems may fall within the scope of the MDR or IVDR and, where the conditions of Article 6 are satisfied, qualify as high-risk AI systems under the AI Act (4–6, 9). Table 1 summarises the principal categories.

**Table 1:** Functional classification of AI use cases in medicine.

Use case	Typical medical role	Regulatory relevance
AI-enabled medical devices	Diagnosis, monitoring, prediction, prognosis, treatment support, or device control	May fall under the MDR or IVDR and become high-risk under the AI Act where the conditions of Article 6 are satisfied.
Clinical decision support	Risk scoring, diagnostic suggestions, alerts, and treatment recommendations	Requires careful consideration of intended purpose, human oversight, automation bias, and workflow integration.
Radiology AI	Image reconstruction, detection, segmentation, triage, diagnosis, and reporting support	Often safety-critical because imaging outputs directly influence diagnostic and treatment pathways.
Pathology AI	Whole-slide analysis, tumour detection, grading, and biomarker quantification	Requires validation across staining methods, scanners, laboratories, and patient populations.
AI triage	Prioritisation of patients, referrals, cases, or urgent findings	May significantly affect access to care, waiting times, and escalation decisions.
Hospital workflow AI	Scheduling, bed management, coding, documentation, and resource allocation	Often administrative in nature, but may become clinically significant where care pathways are affected.
Generative documentation	Drafting clinical notes, summaries, letters, instructions, and reports	Requires human review, factuality verification, privacy safeguards, and clear accountability.
Patient-facing chatbots	Health information, symptom checking, reminders, and self-management support	Create risks of false reassurance, unsafe advice, privacy breaches, and unclear responsibility for outputs.

The potential effect of clinical decision support systems’ outputs on the clinical judgement process needs special consideration because their recommendations, risk estimates, and alarms have the potential to affect clinicians’ decisions and therefore cannot be assessed only in terms of prediction; other considerations need to be taken into account as well (13–16). Radiological and pathological algorithms typically lose their accuracy once they are applied in various imaging devices, laboratories, acquisition procedures, and populations that differ from the training cohort (3, 17–19); therefore, for high-risk systems, data governance under Article 10, appropriate validation under Article 15, and post-market monitoring under Article 72 become important compliance requirements. Triage and prioritisation systems can delay treatment for some patients while over-escalating others, so compliance depends on representative datasets, subgroup performance evaluation, and audit trails (1, 2, 4). Generative and patient-facing systems are the most classification-sensitive: an identical foundation model may attract only the transparency obligations of Article 50 when used administratively yet become regulated medical-device software when it produces patient-specific clinical advice (4, 5, 7, 20, 21); this case is examined in Section 9.

## 4 The regulatory landscape: classification and interacting regimes

**Figure 1:**
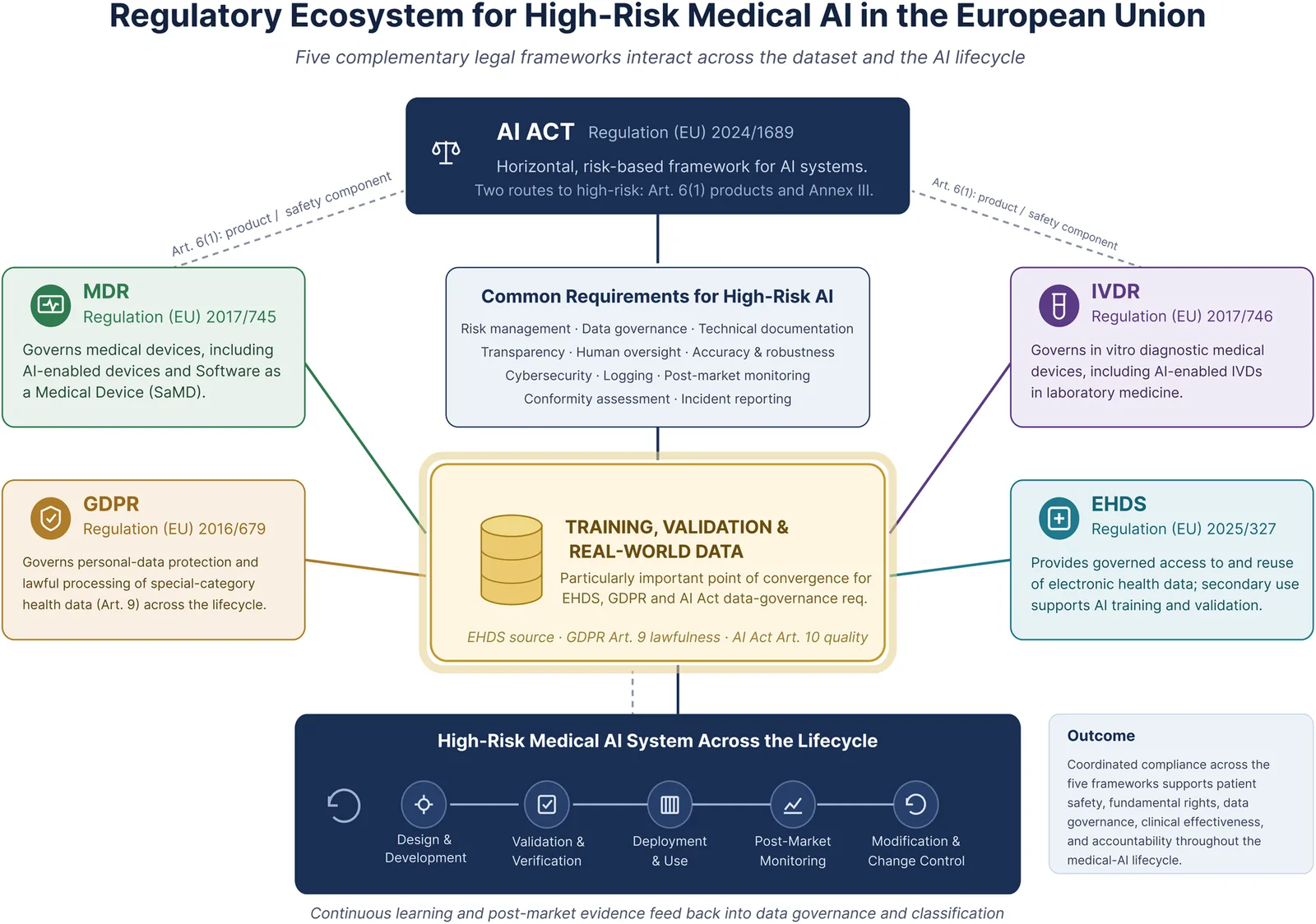
Regulatory ecosystem for high-risk medical AI in the European Union.

Medical AI operates within a regulatory environment in which the AI Act, MDR, IVDR, GDPR, and EHDS apply concurrently. The AI Act introduces an AI-specific layer of governance without replacing sector-specific legislation (4). Figure 1 illustrates their interaction across the AI lifecycle.

### 4.1 Risk-based classification and routes to high-risk

The AI Act classifies AI systems into prohibited practices, high-risk systems, transparency-regulated systems, and minimal-risk systems, while also establishing a separate regime for general-purpose AI (GPAI) models (4, 22). For medical AI, classification depends on intended purpose, clinical role, degree of autonomy, deployment context, and potential impact on patient safety rather than on the underlying machine learning technique.

Article 6 establishes two principal routes to high-risk classification. The product-based route under Article 6(1) classifies an AI system as high-risk where it constitutes a product or a safety component of a product covered by Union harmonisation legislation listed in Annex I and subject to third-party conformity assessment; both the MDR and the IVDR are listed in Annex I (4–6). The second route is established through Annex III, which identifies specific high-risk application areas, including systems used for emergency call assessment or dispatch prioritisation (4). Classification determines the entire governance pathway.

### 4.2 The five regimes and their points of convergence

Table 2 summarises the five frameworks. Their interaction occurs across the lifecycle, with dataset governance providing a particularly important point of convergence because data access, lawfulness of processing, and suitability for the intended purpose are governed by different instruments.

**Table 2:** Interaction between the EU legal regimes governing medical AI.

Regime	Main purpose	Relevance to medical AI
AI Act	AI-specific risk governance and rights protection	Classifies many AI-enabled medical devices and IVDs as high-risk where the conditions of Article 6 are satisfied.
MDR	Safety and performance of medical devices	Determines whether software qualifies as a medical device and which conformity assessment route applies.
IVDR	Safety and performance of in vitro diagnostic medical devices	Applies to diagnostic AI used in laboratory medicine, pathology, genomics, or companion diagnostics.
GDPR	Protection of personal data and data subject rights	Applies where medical AI processes personal, health, genetic, or biometric data.
EHDS	Governance, infrastructure, and standards for health data access and reuse	Supports access to interoperable health data for healthcare delivery, research, innovation, patient safety, and regulatory purposes.

The MDR governs intended purpose, risk classification, clinical evaluation, technical documentation, conformity assessment, CE marking, and post-market surveillance (5). Where a device is also high-risk under the AI Act, additional obligations apply concerning data governance, human oversight, transparency, logging, robustness, and cybersecurity (4, 9). The IVDR performs an analogous function for in vitro diagnostics (6). The GDPR applies because medical AI depends extensively on special-category personal data under Article 9 (7). The EHDS provides a governed mechanism for obtaining representative, multi-institutional training and validation datasets through national Health Data Access Bodies (8).

The interaction among three of these regimes is particularly clear at the level of a single training dataset: the EHDS governs the source of the data and the conditions of access, Article 9 GDPR governs the lawfulness of processing special-category personal data, and Article 10 AI Act governs the resulting dataset’s quality, representativeness, and suitability (4, 7, 8). A dataset may be lawfully obtained under the EHDS and lawfully processed under the GDPR yet still fail Article 10 for insufficient representativeness. Conversely, a technically robust model may prove impossible to deploy lawfully because its underlying data processing lacks a valid legal basis. Treating these obligations as components of a single governance problem is the central analytical premise underlying Stage 3 of the proposed framework (Section 8).

There might be various Article 9(2) GDPR criteria that apply to different Member States; therefore, the dataset governance artefact notes the particular national legal criteria of each participating data source. EHDS secondary use will come into operation progressively and, thus, will gain more importance in relation to medical AI governance.

The AI Act seeks to reduce unnecessary overlap with sector-specific regulation, including by allowing technical documentation to satisfy both AI Act and sectoral requirements (4). Regulation (EU) 2026/1744 strengthens this integrative approach by allowing the Commission, under defined conditions, to limit specific AI Act requirements for Article 6(1) systems where Annex I sectoral legislation provides an equivalent level of protection (23). This supports single-lifecycle governance but does not remove the need to coordinate the AI Act, MDR, IVDR, GDPR, and EHDS.

### 4.3 General-purpose AI models and the medical AI value chain

Many modern medical AI systems rely on GPAI models. The AI Act regulates GPAI models under Articles 51–56 through a regime that is distinct from, but complementary to, the rules for high-risk AI systems. A foundation model without a specific medical intended purpose will generally fall within the GPAI regime and is subject to documentation and downstream-information obligations under Article 53 and Annex XII, with additional obligations for GPAI models posing systemic risk under Article 55 (4).

Where a downstream actor places a GPAI-based system on the market under its own name, gives it a medical intended purpose, or substantially modifies it, that actor assumes the obligations of a provider of a high-risk system under Article 25 (4). Article 25 also requires the upstream GPAI provider to supply the information and documentation the downstream provider needs to discharge its own Article 10 and Article 11 obligations.

A critical practical limitation arises where the upstream provider does not fulfil this obligation—a realistic failure mode, particularly for non-EU foundation model providers. In such cases the downstream provider inherits the full Article 6(1) compliance burden but may have no contractual lever to compel the upstream provider’s documentation. This gap must be addressed at procurement through explicit contractual terms requiring Annex XII documentation as a condition of supply. The procurement recommendation in Section 10 addresses this directly.

Because the upstream model may be retrained or otherwise altered by its original provider, clinically relevant behaviour can change without any action by the deployer. The allocation of monitoring and change-control responsibility between the GPAI provider, the downstream high-risk provider, and the deployer therefore becomes a substantive governance question taken up at Stages 7 and 8 (Sections 8.7–8.8).

## 5 Compliance obligations for high-risk medical AI

Classification as high-risk triggers a comprehensive set of obligations extending well beyond market entry to encompass the entire AI lifecycle. Table 3 summarises the principal obligations. Article 8 permits compliance to be demonstrated through established sector-specific conformity assessment procedures, so providers of AI-enabled medical devices can integrate AI-specific controls within existing MDR or IVDR quality management systems rather than establish parallel processes (4, 9).

**Table 3:** Core compliance obligations for high-risk medical AI.

Obligation	AI Act reference	Medical AI implication
Risk management	Art. 9	Continuous lifecycle risk management addressing clinical, technical, workflow, bias, cybersecurity, misuse, and model drift risks.
Data governance	Art. 10	Documentation of data provenance, representativeness, annotation practices, subgroup performance, bias assessment, and dataset suitability.
Technical documentation	Art. 11	Maintenance of documentation sufficient to support conformity assessment, regulatory review, and lifecycle traceability.
Logging and traceability	Art. 12	Recording of model versions, inputs, outputs, timestamps, user interactions, overrides, and adverse events.
Transparency	Art. 13	Provision of clinically meaningful information regarding intended purpose, limitations, performance characteristics, uncertainty, and appropriate use.
Human oversight	Art. 14	Mechanisms enabling clinicians and other users to understand, question, override, or suspend AI-supported decisions where necessary.
Accuracy, robustness and cybersecurity	Art. 15	Validation across institutions, devices, patient populations, and clinical workflows, together with protection against foreseeable cybersecurity threats.
Quality management	Art. 17	Integration of AI-specific controls into organisational quality management systems spanning design, validation, monitoring, and incident response.
Conformity assessment and CE marking	Arts. 43, 47, 48	Demonstration of compliance through the applicable conformity assessment procedures and preparation of declarations of conformity.
Registration	Art. 49	Registration requirements applicable to relevant categories of high-risk AI systems, particularly certain Annex III applications.
Post-market monitoring	Art. 72	Continuous monitoring of real-world performance, incidents, subgroup outcomes, model drift, software updates, and cybersecurity events.

Viewed alongside existing medical-device legislation, many AI Act duties extend established regulatory processes. Risk management, technical documentation, transparency, quality management, conformity assessment, and post-market monitoring can largely be integrated with MDR/IVDR processes, while accounting for AI-specific hazards such as dataset bias, automation bias, model drift, adversarial manipulation, and algorithmic opacity (4–6, 24). The more distinctive AI Act requirements concern data governance, human oversight, operational resilience, and lifecycle traceability. Article 10 introduces specific requirements for training, validation, and testing datasets and provides a particularly important point of convergence with Article 9 GDPR and the EHDS. Article 14 addresses human oversight, Article 15 addresses accuracy, robustness, and cybersecurity, and Article 12 requires systematic logging throughout the operational lifetime of the system (4, 5).

## 6 Unsettled boundaries at the medical-AI/device-law interface

Two preliminary questions must be resolved before the framework can be applied, neither answered definitively by the legislative text. The first concerns scope: when does clinical decision-support software become a regulated medical device? The second concerns change over time: when does modification of a certified learning system require a new conformity assessment?

### 6.1 When does clinical decision support become a regulated device?

MDCG 2019-11 (Rev. 1, June 2025) asks whether software processes data beyond simple storage or transmission, whether it is intended to benefit individual patients, and whether it has a medical intended purpose within the meaning of Article 2(1) MDR. Rule 11 classifies software informing diagnostic or therapeutic decisions as Class IIa by default, rising to Class IIb where an erroneous decision could result in serious deterioration and to Class III where it could result in death or irreversible deterioration (5, 25). *SNITEM and Philips France* (C-329/16) confirm that software using patient-specific data to identify contraindications, interactions, or excessive dosages may qualify as a medical device, and that intended purpose remains the decisive criterion (26).

The principal difficulty is that the distinction between *informing* and *driving* a clinical decision is expressed through intended purpose, whereas the practical influence of AI systems is behavioural. The automation-bias literature demonstrates that clinicians frequently rely on AI-generated scores, alerts, and recommendations even where they formally retain decision-making authority (13, 27). The AI Act further complicates this by introducing the Annex III exemption for systems that do not materially influence decision-making, creating a threshold related to but distinct from qualification under the MDR (4).

Under a *formalist* interpretation, qualification depends on the intended purpose declared by the manufacturer. This promotes legal certainty but creates a weakness: manufacturers may avoid regulation through disclaimers describing systems as merely advisory even where they materially influence clinical decisions in practice. Under a *functional* interpretation, qualification depends not only on the declared intended purpose but also on the system’s actual capacity to influence individual clinical decisions. This better reflects clinical reality and is more consistent with the modular analysis in *SNITEM* (§40) and with MDCG guidance addressing compound software functionality (25, 26).

On balance, we favour the functional interpretation. A classification approach based only on the manufacturer’s description could under-regulate software whose outputs foreseeably shape individual clinical decisions. Two limits keep this interpretation bounded. First, the software must act on data relating to an individual patient, excluding general reference tools and population-level analytics (26). Second, its influence on clinical decision-making must be reasonably foreseeable in the intended deployment rather than merely hypothetical (13). A worklist tool that reorders patient studies on the basis of suspected pathology may therefore fall within this analysis, whereas a dashboard reporting aggregate throughput would not. Final Commission guidance on Article 6 should be considered when available (22).

### 6.2 When does updating a learning system trigger a fresh conformity assessment?

Medical AI systems evolve through retraining, adaptation, and performance drift. Article 3(23) AI Act defines ’substantial modification’, and Article 43(4) requires a new conformity assessment whenever such a modification occurs. An exception applies where anticipated future modifications have been predefined by the provider during the initial conformity assessment and documented within the Annex IV technical documentation (4).

Several uncertainties remain. The Regulation does not specify the level of detail required to define the predetermined change envelope, and the concept of substantial modification under the AI Act does not correspond directly to any equivalent concept under the MDR: MDCG 2020-3 provides guidance only in relation to transitional provisions for legacy certificates, and MDR change management is addressed primarily through notified body oversight and quality management systems (5, 28).

The most developed external reference point is the FDA Predetermined Change Control Plan (PCCP), formalised in December 2024 (29): manufacturers identify anticipated modifications during initial authorisation, specify how those modifications will be implemented and validated, and assess their anticipated effect on safety and effectiveness. The joint FDA, Health Canada, and MHRA guiding principles published in 2023 require anticipated modifications to be clearly defined, verifiable, risk-based, and consistent with the device’s original intended purpose (30, 31). A cross-sectional study of machine-learning-enabled medical devices authorised by the FDA during 2024 found limited uptake of PCCPs and considerable variation in transparency practices (32), providing a cautionary lesson for European implementation.

Pending harmonised standards or common specifications under Article 43(4), we interpret retraining that alters clinically relevant performance characteristics recorded in the technical documentation as a substantial modification requiring renewed conformity assessment, with AI Act and MDR change assessments undertaken in a coordinated manner.

## 7 The gap in existing frameworks

Existing governance, regulatory, and certification-orientated approaches address important parts of the medical-AI lifecycle, but the literature reviewed here does not provide an equivalent architecture that explicitly reconciles the AI Act, MDR, IVDR, GDPR, and EHDS through linked auditable artefacts across the complete lifecycle of a high-risk medical AI system. The relevant approaches can be grouped into several complementary families.

The first comprises principle-based governance frameworks. The OECD AI Principles (33) and WHO guidance (1, 2) articulate important normative objectives but provide limited guidance on operational compliance and do not offer an implementation pathway within the European legal framework.

The second covers international AI and medical device standards, including the NIST AI RMF (34), ISO/IEC 42001 (35), ISO/IEC 23894 (36), ISO 14971 (24), IEC 62304 (37), and IEC 82304-1 (38). These provide valuable operational controls but remain largely sector neutral and do not address the overlapping obligations that arise simultaneously under all five European instruments.

The third comprises healthcare-specific deployment frameworks. FUTURE-AI (3) offers valuable technical and organisational guidance but does not explain how organisations should coordinate legal obligations arising simultaneously under the five regimes. Within its own domain of technical trustworthiness, FUTURE-AI is more operationally detailed than the present framework; the distinct contribution here is the multi-regime *legal* reconciliation performed at each stage, which FUTURE-AI does not attempt. The FDA PCCP (29) provides a structured mechanism for managing post-deployment modifications but is confined to change control rather than the broader governance lifecycle. Recent work by Barucci et al. (39) proposes a three-phase lifecycle for AI in clinical research, comprising training, real-world testing, and post-marketing monitoring, with regulatory burdens aligned to AI maturity. This provides a complementary lifecycle-based perspective focused on clinical research and ethics-committee evaluation, whereas the present framework addresses cross-regulatory governance across five EU regimes through stage-specific auditable artefacts.

The closest existing work is Aboy, Minssen, and Vayena (9), which provides an authoritative account of how the AI Act applies to AI-enabled medical devices, including classification, the Article 6 high-risk pathway, and provider obligations. That work is primarily interpretative: it explains what the overlapping legal regimes require but does not propose an operational method for implementing those requirements. Its primary focus is the AI Act, MDR, and IVDR, with data protection treated largely as contextual background. Published in April 2024, it also predates the EHDS Regulation and does not operationally address the boundary questions examined in Section 6. The present paper therefore extends this earlier analysis from legal interpretation towards practical implementation. A further strand of recent work addresses the translation of Responsible AI and regulatory requirements into medical-device certification and engineering processes. Mallardi et al. (40) propose an extended MLOps approach intended to support Responsible AI and the certification of machine-learning models as medical devices. Related work by Rosmarino et al. (41) examines the automated generation of regulatory documentation within MLOps pipelines for AI-based medical software. These approaches are important because they move beyond high-level principles towards implementation within development and certification workflows. Their primary focus, however, is the engineering and documentation infrastructure supporting medical-device compliance rather than the reconciliation of the AI Act, MDR, IVDR, GDPR, and EHDS as interacting legal regimes. The present framework is therefore complementary: it specifies the cross-regulatory governance logic and evidence relationships that such engineering pipelines could subsequently implement. Table 4 summarises the comparison.

**Table 4:** Comparison of the proposed compliance-by-design framework with selected AI governance frameworks.

Framework	Primary scope	Multi-regime EU integration	Full lifecycle coverage	Operational artefacts
WHO AI for Health	Ethical and governance principles for healthcare AI	No	Partial	No (principles rather than processes)
FUTURE-AI	Trustworthy and deployable healthcare AI	No	Partial	Partial (detailed technical/organisational guidance)
OECD AI Principles	Human-centred and trustworthy AI governance	No	No (normative principles, not implementation processes)	No
FDA PCCP	Adaptive AI and post-market modification	No (single legal instrument)	No (change control only)	Yes (change control only)
Aboy, Minssen & Vayena (9)	AI Act and MDR/IVDR analysis	Partial (AI Act, MDR, IVDR; GDPR contextual; predates EHDS)	Partial (provider obligations, not staged lifecycle governance)	No (interpretative analysis)
Mallardi et al. (40)	Responsible AI and MLOps support for medical-device certification	Partial; certification-oriented rather than five-regime reconciliation	Partial; development, compliance, and monitoring processes	Partial; structured compliance and documentation within MLOps
Proposed framework	Lifecycle governance of high-risk medical AI	Yes (AI Act, MDR, IVDR, GDPR, EHDS)	Yes	Yes (auditable lifecycle artefacts)

The distinguishing feature is not lifecycle staging but the reconciliation of all five European regimes at each stage through auditable artefacts. Entries describe relative emphasis and are representative rather than exhaustive; FUTURE-AI is more detailed on technical trustworthiness within its own scope.

The framework also draws on a distinct theoretical lineage from the governance literature reviewed above. Regulation-by-design scholarship argues that regulatory objectives are more reliably achieved when compliance requirements are embedded into the structure of a system or process rather than imposed as external, retrospective controls (10). Meta-regulatory and enforced-self-regulation theory holds that where a regulator cannot feasibly prescribe every operational detail, the more effective role is to require regulated actors to produce their own auditable evidence of compliance, which the regulator then reviews rather than replicates (11). Risk-based regulation theory adds that oversight intensity should scale with the risk a system poses rather than applying uniform scrutiny (42). The eight-stage model operationalises these three principles jointly: compliance is embedded at each design and deployment decision (regulation by design); the artefact produced at each stage is the auditable evidence a notified body or regulator inspects rather than replicates (meta-regulation); and the depth of each artefact—particularly Stages 3 through 5—scales with the clinical risk established at Stage 1 (risk-based regulation). Positioning the framework within this literature clarifies that its contribution is not only doctrinal synthesis but an application of established regulatory-theoretic principles to the specific coordination problem created by five overlapping EU legal regimes.

Existing scholarship provides complementary legal, governance, certification, and technical approaches to medical AI. Building on these strands, the present framework focuses specifically on translating the interacting obligations of the AI Act, MDR, IVDR, GDPR, and EHDS into linked lifecycle artefacts that make their cross-regulatory relationships inspectable. Stage 3 illustrates the point concretely: a generic lifecycle model records that data governance precedes development, whereas the stage proposed here records which instrument governs the source of the data (EHDS), which establishes the lawfulness of its processing (Article 9 GDPR), and which determines its quality and representativeness (Article 10 AI Act), resolving the three into a single suitability justification that a notified body can inspect.

## 8 A medical AI compliance-by-design model

The regulatory obligations analysed in Section 5 are translated into proposed governance practices spanning design, validation, deployment, monitoring, and continuous improvement. Compliance by design integrates legal, ethical, and patient-safety requirements throughout the AI lifecycle rather than treating compliance as an end-of-development activity (2, 4, 9). The term is used deliberately to extend the data-protection-by-design logic of Article 25 GDPR (7) from data protection alone to classification, validation, oversight, conformity assessment, and change control across all five regimes.

**Figure 2:**
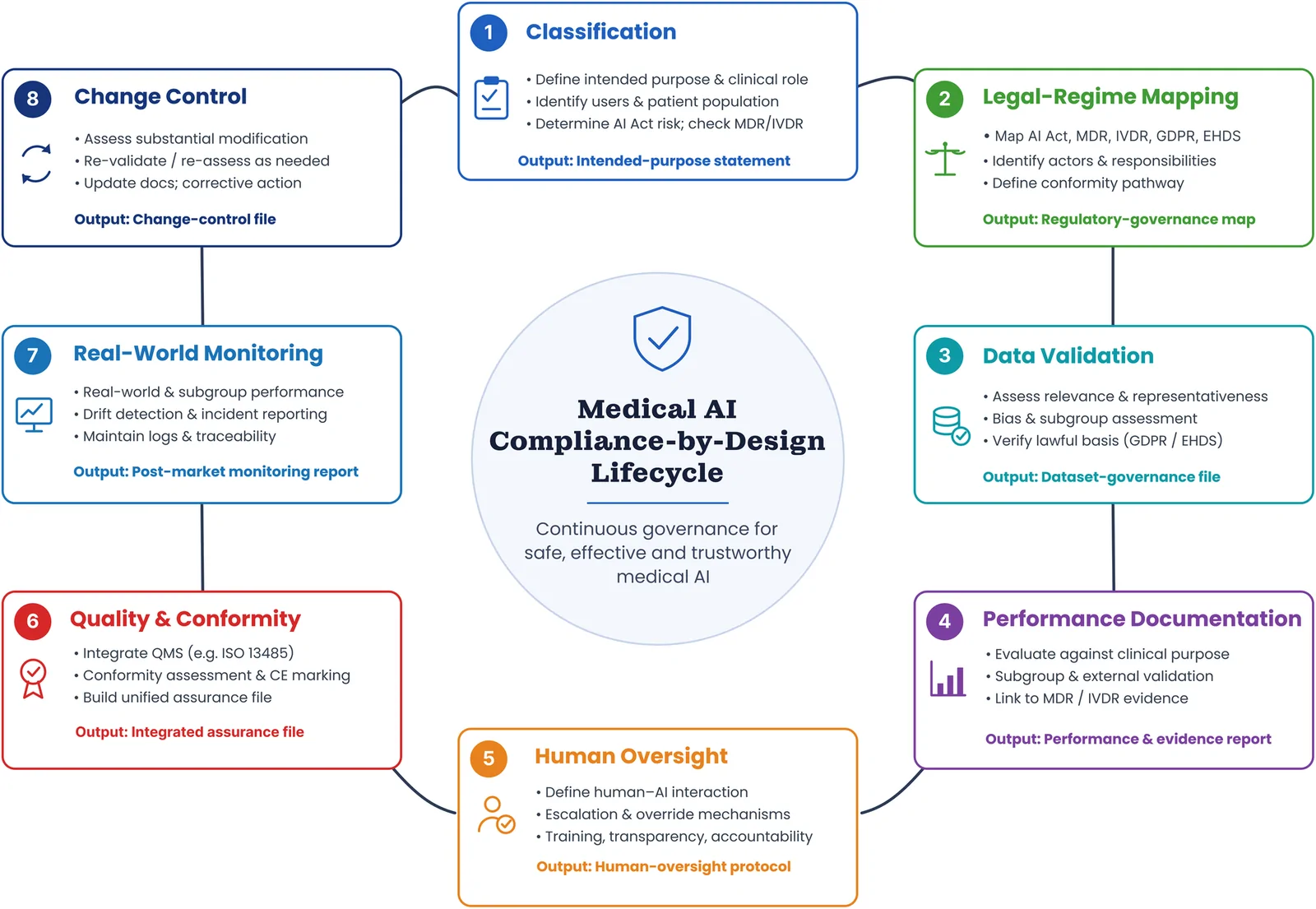
Medical AI compliance-by-design lifecycle.

The model is cyclical rather than linear because changes in intended purpose, datasets, or real-world performance may require renewed classification, validation, or change control. Figure 2 summarises the lifecycle.

As argued in Section 7, the novelty lies in translating legal obligations into linked governance artefacts that jointly instantiate regulation-by-design, meta-regulatory, and risk-based principles, rather than in the lifecycle staging itself. The artefacts are *chained*: the intended-purpose statement produced at Stage 1 fixes the legal framework mapped at Stage 2, which in turn scopes the dataset governance file at Stage 3, and so on through to the change-control file at Stage 8. Three artefacts are shown as worked excerpts, all completed for the patient-facing triage configuration discussed in Section 9: the Stage 2 regulatory governance map (Table 5), the Stage 3 dataset governance file (Table 6), and the Stage 8 change-control file (Table 7).

**Table 5:** Worked excerpt of the Stage 2 regulatory governance map, completed for the patient-facing triage configuration of Section 9 (Configuration B).

Field	Illustrative entry
AI Act status	High-risk via Article 6(1) (Annex I product route, MDR); the Article 50 transparency duty to disclose AI interaction also applies to the patient-facing channel.
MDR/IVDR	MDR applies; under Rule 11 the patient-specific triage function sits at Class IIb on the illustrative facts; IVDR not engaged.
GDPR role and lawful basis	Deploying organisation acts as controller; special-category processing relies on the applicable Article 9(2) national condition, recorded per source at Stage 3; Article 22 (solely automated decisions) assessed at Stage 5.
EHDS position	Developer/deployer acts as data user for secondary-use training and validation data (permit and secure processing environment); prospective where national mechanisms are not yet operational.
Article 25 value chain	Upstream GPAI provider supplies Annex XII documentation; by giving the model a medical intended purpose the deployer becomes provider of a high-risk system and inherits the Article 10 and Article 11 duties.
Responsible actors	Deployer-as-provider; upstream GPAI provider; notified body; healthcare deployer; controller and any processors.
Conformity pathway	MDR (Annex I) conformity assessment used, under Article 8, to demonstrate AI Act high-risk compliance through a single procedure; notified body involved.
Cross-references	Feeds the Stage 3 dataset governance file, the Stage 5 Article 22 assessment, and the Stage 6 integrated assurance file.

Entries chain to the Stage 3 dataset governance file (Table 6) and the Stage 8 change-control file (Table 7). Device-class indications are illustrative and not a classification determination for any specific product.

**Table 6:** Worked excerpt of the Stage 3 dataset governance file, completed for the patient-facing triage configuration of Section 9.

Field	Illustrative entry
Data provenance	Primary-care encounter records and triage-outcome data from four regional health systems, accessed for secondary use.
Access route (EHDS)	Data permit issued by the national Health Data Access Body; access confined to a secure processing environment; permitted purpose limited to training and evaluation of the triage model.
Lawful basis (Art. 9 GDPR)	Processing of special-category health data justified under the applicable Article 9(2) condition relied on in national law, recorded for each contributing source; pseudonymisation applied within the secure processing environment.
Inclusion/exclusion	Adults presenting with undifferentiated symptoms; paediatric and emergency-pathway encounters excluded and recorded as out-of-scope limitations.
Subgroup representation	Age, sex, language of consultation, and comorbidity burden tabulated against the intended deployment population; under-representation of non-native-language consultations flagged.
Annotation methodology	Triage outcomes derived from documented clinical disposition; ambiguous dispositions adjudicated by a second reviewer; inter-rater agreement recorded.
Bias and residual gap	Residual discrepancy in the representation of non-native language and rural cases documented; mitigation was deferred to data collection and recorded as a known limitation for Stage 4 validation and Stage 7 monitoring.
Suitability justification	Linking statement that connects provenance, legal basis, and representativeness to the purpose that was set at Stage 1, leaving unresolved gaps unaddressed.

The EHDS access route shown is prospective where national secondary-use mechanisms are not yet operational; the same fields are completed against the national route relied on in the interim.

**Table 7:** Worked excerpt of the Stage 8 change-control file, completed for the patient-facing triage configuration of Section 9 for the case in which the upstream foundation model is retrained by its original provider.

Field	Illustrative entry
Modification	Upstream GPAI provider retrains the foundation model; downstream triage ranking and escalation behaviour shift without local action by the deployer.
Classification of change	Assessed against Article 3(23) and the predefined change envelope documented in Annex IV; under the interpretation adopted in Section 6.2, the change is treated as substantial because clinically relevant performance characteristics are affected.
Affected components	Triage ranking outputs, escalation thresholds, and confidence calibration; instructions for use.
Validation evidence	Re-validation on held-out and subgroup test sets; comparison against the pre-change performance baseline recorded at Stage 4; regression on safety-critical presentations.
Regulatory action	On that interpretation, renewed conformity assessment is coordinated across the AI Act (Art. 43(4)) and the MDR change-management route; notified body notified; technical documentation updated.
Risk and safety	Updated Article 9 risk assessment; interim mitigation and documented rollback to the last validated version pending reassessment.
User communication	Deployer notified per the procurement contract’s upstream-change clause; affected sites informed; revalidation status disclosed.
Monitoring linkage	Post-change monitoring plan feeding Stage 7; new model version, deployment date, and outcome of post-update evaluation recorded.

The entries close the artefact chain begun in Tables 5 and 6.

The proposed artefacts are not intended to create eight parallel controlled documents. Here, an “artefact” denotes a controlled body of evidence or cross-regulatory view that may be implemented as a new record, an extension of existing QMS or technical documentation, or an index linking evidence already maintained under the MDR/IVDR and standards such as ISO 13485, ISO 14971, IEC 62304, and ISO/IEC 42001. In practice, Stage 1 can be incorporated into intended-purpose and technical documentation; Stage 4 can draw on existing clinical or performance evaluation and validation evidence; Stage 7 can extend existing post-market surveillance records; and Stage 8 can extend software and QMS change-control records. Stages 2, 3, and 5 provide cross-regulatory views where existing records do not fully capture regulatory responsibility allocation, combined AI Act/GDPR/EHDS data-governance evidence, or AI-specific human-oversight requirements. Stage 6 functions primarily as an assurance index linking these controlled records. The aim is therefore to reconcile and reuse existing evidence rather than duplicate it.

### 8.1 Stage 1: classify by intended purpose and clinical role

*Guiding question:* What are the system’s intended purpose, clinical role, target users, and intended patient population?

Classification should precede final system development because it determines the applicable legal framework, validation requirements, instructions for use, and post-market obligations. It should be based on the system’s actual and reasonably foreseeable use rather than solely on the manufacturer’s marketing description or product labelling (4–6).

*Output:* An intended-purpose statement specifying the medical purpose, target users, intended patient population, deployment setting, input data, output type, degree of automation, expected user actions, and known limitations.

### 8.2 Stage 2: map the applicable legal regimes

*Guiding question:* Which European legal frameworks apply, and which actors are responsible for compliance?

The applicable AI Act, MDR, IVDR, GDPR, and EHDS obligations are identified according to the system’s architecture, intended purpose, data flows, and deployment context, together with the responsibilities of providers, deployers, manufacturers, importers, distributors, controllers, and processors, including Article 25 allocation of provider obligations where a GPAI-based system is given a medical intended purpose downstream (4–6).

*Output:* A regulatory governance map documenting the applicable legal frameworks, regulatory obligations, responsible actors, accountability relationships, and triggers for reassessment following material regulatory change (7, 8). Table 5 gives a worked excerpt.

### 8.3 Stage 3: validate data before development and deployment

*Guiding question:* Are the datasets suitable, lawful, representative, and appropriately assessed for potential bias?

Article 10 AI Act, together with the data governance requirements of the GDPR and the EHDS, requires confirming the clinical relevance and representativeness of the data, identifying under-represented groups and subgroup disparities, assessing annotation quality and label reliability, detecting institutional or technical artefacts, and establishing a lawful basis for processing special-category personal data under Article 9 GDPR. Where datasets are obtained through the EHDS secondary-use framework, the applicable data permit, the responsible Health Data Access Body, the secure processing environment, the anonymisation or pseudonymisation status, and the permitted-purpose scope should all be documented (4, 7, 8). Data quality assessment should be conducted in accordance with the ISO/IEC 5259 series (43). This stage is a particularly important point of convergence among the three regimes: EHDS access to the data source, Article 9 GDPR lawfulness of processing, and Article 10 AI Act requirements for quality and representativeness.

The dataset governance file should record the specific Article 9(2) condition relied upon for each source together with its national legal basis, since Article 9(2) does not itself authorise processing of health data for AI development but permits Member States to establish the relevant legal conditions (7). The Digital Omnibus on AI reinstates a strict-necessity threshold for processing special-category data for bias detection and correction, so bias-mitigation activities relying on sensitive attributes must be justified against that threshold and documented accordingly (7, 23).

*Output:* A dataset governance file documenting data provenance, collection context, lawful basis, EHDS permit details, inclusion and exclusion criteria, annotation methodology, missing-data handling, bias assessment, subgroup representation, privacy safeguards, and a justification of dataset suitability. Table 6 gives a worked excerpt.

### 8.4 Stage 4: document model performance and clinical evidence

*Guiding question:* Does the system perform safely and effectively for its intended clinical purpose?

Performance evaluation under Article 15 should be aligned with the system’s intended purpose rather than generic benchmark performance and linked to the applicable MDR or IVDR clinical performance requirements. Results should be reported using recognised frameworks such as FUTURE-AI, CONSORT-AI, and DECIDE-AI (3, 14, 15). For generative and patient-facing outputs, the QUEST criteria provide a structured basis for evaluating quality, usability, factuality, and safety. Performance documentation should, where available, include evidence of clinical effectiveness in the intended deployment setting rather than regulatory metrics alone.

*Output:* A performance and clinical evidence report documenting performance metrics, subgroup analyses, validation results, recognised limitations, uncertainty thresholds, and known contraindications.

### 8.5 Stage 5: define human oversight and accountability

*Guiding question:* Can users meaningfully supervise, interpret, challenge, and override the system?

Within the proposed framework, Article 14 human-oversight requirements are implemented through appropriate interfaces, user training, escalation policies, override controls, and accountability processes tailored to the relevant use case (4). For example, for a radiology system, compliance may involve visual explanation tools and confidence measures; for triage systems, it will involve escalation thresholds and audit trails; for generative clinical documentation, it may require a review-and-approval workflow; and for patient-facing conversational systems, it may require a mandatory human escalation route.

Article 14 of the AI Act must also be considered alongside Article 22 GDPR, which protects individuals from certain decisions based solely on automated processing that produce legal or similarly significant effects (7). Formal human involvement may not be sufficient where, in practice, a clinician routinely defers to the AI-generated decision (13). The oversight protocol should therefore address both the human-interface and override requirements of Article 14 and the separate assessment required under Article 22.

*Output:* An overview of the human-oversight protocol, including user roles, training, escalation criteria, override procedures, documentation and accountability procedures, as well as Article 22 assessment.

### 8.6 Stage 6: integrate quality management, conformity assessment, and documentation

*Guiding question:* How are AI Act obligations integrated with existing MDR and IVDR procedures?

Article 17 and Articles 43 to 48 are best enforced using an integrated assurance framework. Article 43(3) of the AI Act explicitly provides for the application of current sectoral conformity assessment schemes in Annex I to meet the specific AI requirements within medical devices’ quality management systems (4). IEC 62304 lays out the lifecycle requirements for medical device software, whereas ISO/IEC 42001 sets requirements for AI Management Systems (35, 37).

*Output:* An integrated assurance file that indexes and cross-references existing controlled evidence, supplemented where a regulatory gap remains, including the intended-purpose statement, regulatory governance map, dataset governance file, performance and clinical evidence, risk-management documentation, human-oversight documentation, cybersecurity documentation, instructions for use, conformity and CE-marking documentation, registration review, and post-market surveillance records.

### 8.7 Stage 7: monitor real-world performance

*Guiding question:* Does the system remain safe and effective after deployment?

For implementation within the proposed framework, Article 72 post-market monitoring is organised across three areas: technical, clinical, and organisational monitoring. Technical monitoring includes input drift, output distributions, confidence calibration, error rates, latency, security issues, and software updates. Clinical monitoring includes false positive and false negative rates, late diagnosis, adverse effects, subgroup analysis, and the physician’s practice of overriding the algorithm. Organisational monitoring includes adherence to instructions for use, off-label use, staff training, and the implementation process. Monitoring responsibilities should be explicitly allocated between providers and deployers through procurement and governance arrangements (4).

*Output:* A real-world performance dashboard and monitoring report that continuously informs risk management, quality management, and future system updates.

### 8.8 Stage 8: update controls and manage change

*Guiding question:* How are incidents, model drift, system modifications, regulatory changes, and corrective actions managed throughout the AI lifecycle?

The change management process needs to differentiate among routine maintenance, adjustment, retraining, use-intent change, and fundamental changes in order to determine when the above actions need internal scrutiny, revalidation, notification of the users, revision of documentation, conformance checking, or regulatory reporting (Articles 3(23) and 43(4)). Serious occurrences will warrant an investigation and regulatory reporting, whereas an unacceptable level of residual risk will require that the system be suspended, restricted, or otherwise corrected (4). The above lifecycle is compatible with IEC 62304, where controlled modification, impact analysis, verification, and validation are mandated throughout the software lifecycle (37). The accountability records generated above, along with the regime map of Stage 2, provide the documentation upon which product liability assessment could follow. A material change in applicable legislation, regulatory guidance, or harmonised standards should also trigger reassessment of the Stage 2 regulatory governance map and, where relevant, revision of affected downstream artefacts.

*Output:* A change-control and corrective-action file documenting the reason for each modification, affected system components, validation evidence, updated risk assessments, user communications, regulatory implications, deployment dates, rollback procedures, and post-update evaluation results. Table 7 gives a worked excerpt.

### 8.9 Requirements-to-artefact traceability

Table 8 makes traceability explicit by linking each lifecycle stage to its regulatory sources, principal responsible actors, governance artefact, and downstream lifecycle implications.

**Table 8:** Requirements-to-artefact traceability crosswalk.

Stage	AI Act provisions	Sector standards/reporting frameworks	Artefact/actor/lifecycle link
1. Classification	Art. 6; Annex III	MDR Art. 2(1), Rule 11 (Annex VIII); MDCG 2019-11	Intended-purpose statement; provider/manufacturer; → Stages 2–8
2. Regime mapping	Arts. 6, 16, 25 (value chain), 50	GDPR Arts. 4, 9; EHDS (data-user provisions)	Regulatory governance map; relevant regulated actors; → Stages 3–8
3. Data governance	Art. 10	GDPR Arts. 9, 35 (DPIA); EHDS Ch. IV; ISO/IEC 5259	Dataset governance file; provider/controller/data user; → Stages 4, 6–8
4. Validation	Art. 15	MDR Annex XIV/IVDR Annex XIII; CONSORT-AI, DECIDE-AI, CLAIM, QUEST, FUTURE-AI	Performance and clinical evidence report; provider/manufacturer; → Stages 5–8
5. Human oversight	Art. 14	GDPR Art. 22	Human-oversight protocol; provider/deployer; → Stages 6–8
6. Quality and conformity	Arts. 17, 43, 47, 48	ISO 13485, ISO 14971, IEC 62304, IEC 82304-1, ISO/IEC 42001	Integrated assurance file; provider/manufacturer; notified-body review where applicable; → Stages 7–8
7. Monitoring	Arts. 72, 73	MDR Arts. 83–86 (PMS, PSUR); ISO/IEC 42001 Annex A	Post-market monitoring report; provider/deployer; → Stage 8 and reassessment where required
8. Change control	Arts. 3(23), 43(4)	MDCG 2020-3; FDA PCCP; IEC 62304 §6–§9	Change-control file; provider/deployer; → earlier stages as affected

Each stage is mapped to its principal regulatory sources, supporting standards, governance artefacts, responsible actor(s), and downstream lifecycle link. Artefacts may reuse or extend existing controlled evidence.

## 9 Worked regulatory application of the compliance-by-design model

This section applies the proposed framework to two constructed regulatory use cases to illustrate its internal logic and cross-regulatory traceability. The examples illustrate how the stages and artefacts can be applied to contested regulatory questions, but they do not establish the framework’s completeness, usability, inter-assessor consistency, regulatory adequacy, or effectiveness in improving compliance or clinical outcomes. For each case, the illustrative assessment considers three traceability checks: whether the integrated framework identifies additional regulatory obligations, clarifies responsibility allocation, and propagates relevant findings across later lifecycle stages.

### 9.1 Case one: one foundation model, two configurations

The first system is a large-language-model-based clinical tool deployed in two configurations sharing the same underlying foundation model:

- **Configuration A**: Documentation assistant for the administration to write notes, discharge, and referral letters using the information provided by clinicians.
- **Configuration B**: Patient-side assistant to help assess symptoms, assist in triaging and self-management.

Table 9 delineates the application over all eight governance phases. Although both configurations use the same foundation model, they diverge at three governance points: classification, evidence and oversight, and change control.

**Table 9:** Stage-by-stage application of the proposed compliance-by-design framework to the same foundation model deployed in two different clinical configurations.

Stage	Administrative documentation assistant	Patient-facing triage assistant	Key governance insight
1. Classification	No medical intended purpose; unlikely to qualify as SaMD	Patient-specific triage; likely SaMD and high-risk	Identical models may receive different regulatory classifications depending on intended purpose.
2. Regime mapping	GDPR and AI Act transparency obligations (Art. 50)	AI Act, MDR, GDPR, and EHDS (where applicable); Art. 25 value-chain position recorded	Applicable obligations depend on clinical role, and provider duties may pass downstream under Art. 25.
3. Data governance	Training-data provenance and documentation	Representative datasets, subgroup coverage, hallucination and omission risk (see Table 6)	Evidence requirements increase with clinical risk.
4. Performance and evidence	Drafting quality and factual accuracy	Clinical accuracy, escalation performance, and patient-safety metrics (QUEST)	Performance evaluation must reflect intended clinical use.
5. Human oversight	Clinician review before incorporation into the medical record	Escalation procedures, uncertainty handling, and AI disclosure to patients	Oversight mechanisms vary according to deployment context.
6. Quality and conformity	Documentation, version control, and transparency	Integration within MDR/AI Act quality management and conformity assessment	A single governance framework supports compliance across multiple regulations.
7. Monitoring	User feedback and documentation quality	Hallucinations, overrides, factuality drift, and safety incidents	Post-market monitoring should reflect real-world deployment risks.
8. Change control	Version control and update documentation	Assessment of substantial modification and provider-managed updates	Compliance is a continuous lifecycle process rather than a one-time exercise.

**Classification and regime mapping.** Configuration A is an administration tool and has no intended purpose as a medical device according to Article 2(1) MDR. It is subject primarily to the GDPR and AI Act transparency provisions (Article 50). Configuration B provides patient-specific symptom assessment and triage. According to MDCG 2019-11 and Rule 11 MDR, it probably has to be considered medical-device software belonging to Article 6(1) high-risk class. The two configurations thus get different classifications, although their model architecture is the same, since the classification criteria are related to intended purpose rather than the design itself. As a traceability check, the Stage 2 regime map identifies two requirements for Configuration B beyond a one-instrument MDR analysis. First, following the value-chain position of Article 25, the institution providing the model with medical intended purpose is a provider of a high-risk system and thus depends on the GPAI provider to provide it with documents satisfying Article 10 and Article 11 requirements. Second, EHDS secondary-use requirements apply to the training dataset.

**Evidence and oversight.** Contrary to Configuration A, Configuration B has to use representative datasets, including subgroup analysis and full compliance with Article 10. Performance evaluation should focus on factual accuracy, omission rates, and escalation behaviour rather than benchmarks in view of WHO warnings about the safety of the output of generative systems (20, 21). Configuration A requires clinician review before AI-generated content enters the medical record; Configuration B requires escalation procedures, uncertainty handling, and disclosure to patients that they are interacting with an AI system.

**Change control.** The underlying foundation model may be retrained by its original provider, so clinically relevant behaviour can change without any action by the deployer. Updates that alter clinically relevant performance characteristics may constitute substantial modifications requiring renewed conformity assessment for Configuration B, as worked through in Table 7; Configuration A’s lower-risk status means equivalent updates typically require only version control and documentation.

### 9.2 Case two: a contested radiology triage tool

Consider an AI system that analyses non-contrast head CT examinations and reprioritises the radiology worklist by flagging studies with suspected intracranial haemorrhage for earlier review. The manufacturer describes the system as a workflow-prioritisation tool: it does not alter the image, issues no diagnosis, and leaves every study to be read in full by a radiologist.

Worklist-prioritisation tools that flag suspected intracranial haemorrhage or large-vessel occlusion on CT are CE marked and in routine clinical use in several European health systems, and their status as medical-device software has been settled in practice. The analysis below abstracts from any particular product and uses the device class to test the framework against a qualification question the legislative text leaves open.

A purely formal reading of Rule 11 and MDCG 2019-11 might accept the manufacturer’s workflow-tool characterisation. The AI Act Annex III material-influence criterion points the other way because reprioritising which patients are reviewed first plainly affects the timing of care and, where a haemorrhage is missed or de-prioritised, the clinical outcome (4, 17). A single-instrument MDR analysis applying the formalist reading of Rule 11 might therefore reach the wrong result.

Under the functional interpretation adopted in Section 6.1, Stage 1 resolves the contested classification by considering the system’s actual and reasonably foreseeable clinical use. Namely, while the flag that signals potential haemorrhage may not be associated with any diagnosis, it conveys meaningful medical information regarding a particular patient, and its foreseeable use consists of the dependence of radiologists on the result it generates, which is a common scenario according to the literature on automation bias (13). On this interpretation, the flag fulfils a medical purpose within Article 2(1) MDR and would therefore qualify as medical-device software under Rule 11. Given the safety-critical consequence of a delayed haemorrhage diagnosis, it sits towards the higher end of the Rule 11 classification and falls within the Article 6(1) high-risk category. Stage 2 maps the system to the AI Act, the MDR, and the GDPR together. Stage 3 surfaces the safety consequence directly: a dataset that under-represents the presentations in which the flag is least reliable creates a foreseeable harm that representativeness and subgroup analysis must address before deployment.

The lesson of the second case is methodological: where the legislative text leaves the boundary open, the framework forces the contested question to be answered explicitly, on the basis of foreseeable clinical use, and documents the answer in artefacts that can be challenged rather than allowing the question to be settled silently by a manufacturer’s label.

Taken together, the two applications illustrate the three traceability checks defined above. In Case One, the integrated analysis identifies the Article 25 value-chain position and EHDS secondary-use obligations and allocates responsibilities across upstream and downstream actors; in Case Two, it makes the contested classification explicit and propagates its consequences into data governance and subsequent lifecycle controls. Because both cases are constructed rather than drawn from the documented regulatory file of a specific certified system, anchoring at least one application in public regulatory documentation is identified in Section 13 as a priority for the next phase of evaluation.

## 10 Actionable recommendations

The framework’s obligations are placed on five groups whose responsibilities are sequential rather than autonomous; effective governance relies on each transition being clearly defined. Table 10 sets out the priority actions and rationale for each group. Three analytical points warrant emphasis beyond the table. First, for developers and providers, regulatory classification is a design decision rather than a downstream compliance step because the intended purpose determines which obligations apply; it must be fixed first, and the data-governance, change-control, and oversight requirements then follow. Second, for healthcare deployers, procurement is the principal governance checkpoint—the last point at which a deployer can verify that a legally compliant system is also safe in its particular setting before patients are exposed. This matters most where the system is built on a foundation model that the provider may retrain outside the deployer’s control: the contract should specify how upstream changes are notified and revalidated, and should require Annex XII documentation as a condition of supply, since without it the downstream provider cannot construct compliant technical documentation under Articles 10 and 11. Third, for policymakers, the disproportionate burden on smaller developers is best addressed not by exemption, which would create a two-tier safety standard, but by shared compliance infrastructure of the kind itemised in Table 10. Notified bodies and regulators should apply the qualification tests by reference to a system’s actual clinical influence rather than its formal description, consistent with the functional reading defended in Section 6 and illustrated in Case Two.

**Table 10:** Actionable recommendations by stakeholder.

Stakeholder	Priority actions	Implementation rationale
Developers and providers	Establish system classification by intended purpose from the outset of design; bring AI Act, GDPR, and EHDS data governance requirements into a single coordinated framework; set predetermined change envelopes early; fold AI controls into existing quality and risk management processes rather than treating them separately.	Intended purpose drives the compliance obligations that apply, and joining up governance across frameworks cuts duplication while keeping the full lifecycle in view.
Healthcare deployers and organisations	Make procurement the main governance checkpoint: demand evidence of validation, human oversight, and post-deployment monitoring before any purchase goes ahead; verify that the intended population and clinical setting match local conditions; secure a written agreement that allocates monitoring and reporting duties between deployer and provider, including notification obligations when the provider updates the underlying model; make Annex XII documentation a contractual condition of supply.	A system that is legally compliant on paper can still pose patient safety risks if used outside its validated scope or if provider-side model changes are not flagged after go-live.
Notified bodies and regulators	Base high-risk and MDR qualification decisions on how a system actually influences clinical decisions, not on how it is formally described; issue practical guidance on managing post-market changes; coordinate assessments across regulatory authorities to avoid inconsistent outcomes.	Consistent functional interpretation reduces regulatory uncertainty and closes the interpretive gap that currently sits between the MDR and AI Act qualification tests.
Health Data Access Bodies and data holders	Establish unambiguous permissions covering AI development and validation use cases; attach provenance and representativeness metadata in a format that maps directly onto the Stage 3 compliance artefact.	Sound data access governance underpins Article 10 compliance and raises dataset quality across the development lifecycle.
Policymakers and standards bodies	Commission harmonised standards for Arts. 10, 14, and 15; reconcile conflicting definitions of “substantial modification” across regulatory regimes; give SMEs access to shared compliance infrastructure, including template artefacts, pre-validated reference datasets through Health Data Access Bodies, risk-proportionate fees, and pooled notified-body capacity; use Digital Omnibus provisions to remove redundant requirements for Annex I products; tie conformity assessment to health technology assessment processes.	Workable implementation depends on coherent standards, regulation that is proportionate enough not to shut out smaller innovators, and a credible link to demonstrated real-world clinical benefit.

## 11 Discussion: implementation challenges

Coordination of regulatory requirements throughout the entire AI lifecycle, rather than interpretation of any specific legal instrument, represents the key challenge in implementing medical AI governance. Technical, clinical, organisational, and regulatory requirements have to be addressed simultaneously; the proposed eight-stage framework is intended to provide a structured way to coordinate them.

Implementation of Article 10 implies more than just lawful data access and includes training and validation datasets representative of intended patients and deployment environments. Retrospective clinical datasets tend to be under-represented in relevant patient groups or reflect particular institutional procedures, which makes it necessary to address the issues of bias and representativeness through continuous governance rather than one-off development activities (4, 17). Internal performance tends not to generalise across hospitals, imaging devices, laboratory procedures, or patient cohorts, which makes robust external validation and continuous post-market assessment critical (14). Human oversight is much more complex than legal compliance; proper user training, integration into workflow, user interfaces, and tools for managing automation bias are required to enable meaningful clinician control (4, 13). Cybersecurity and post-market change management also require continuous efforts as clinical practice, datasets and deployment environments keep changing (4, 34). Smaller companies might face disproportionately high compliance costs due to the necessity of engaging with all five instruments simultaneously (9, 44).

More broadly, there is an issue of the relationship between regulatory compliance and clinical value. While the AI Act, along with MDR and IVDR, determines whether an AI system is safe enough, transparent and effectively governed to be deployed, it does not say anything about clinical benefits or the effectiveness of the use of healthcare resources. Future medical AI governance has to be complemented by health technology assessment, real-world evidence generation, economic evaluation, and patient-centred outcome measurements. Thus, the core question regarding medical AI will evolve from its legal compliance to its clinical effectiveness.

## 12 Note on the legislative timetable

The AI Act entered into force on 1 August 2024 and is being implemented through a phased timetable. Prohibited AI practices, relevant definitions, and AI-literacy provisions became applicable on 2 February 2025, followed by governance provisions and GPAI-model obligations on 2 August 2025. Most remaining provisions became applicable on 2 August 2026, subject to the revised timetable for high-risk AI systems (22). Regulation (EU) 2026/1744 (Digital Omnibus on AI), which entered into force on 27 July 2026, subsequently amended that timetable. Under the revised framework, the relevant high-risk requirements apply from 2 December 2027 for high-risk systems classified under Article 6(2) and Annex III, and from 2 August 2028 for high-risk systems classified under Article 6(1), including qualifying AI-enabled medical devices and in vitro diagnostic medical devices (23).

The Omnibus also introduces targeted amendments, including clarification of the concept of “safety component” and measures to reduce overlap between the AI Act and sector-specific legislation such as the MDR and IVDR. It also addresses the processing of special categories of personal data for bias detection and correction, subject to a strict-necessity standard and safeguards, as discussed at Stage 3 (23). Supporting Commission guidance and harmonised standards continue to develop and should therefore be checked at the point of application. Such regulatory developments do not alter the framework’s overall lifecycle logic, but material changes should trigger reassessment of Stage 2 and any affected downstream artefacts.

## 13 Limitations and future validation

There are three limits arising from the conceptual nature of the paper. Firstly, the framework has yet to be tested against outcomes of any kind or any deployment data. Secondly, the worked examples in Section 9 illustrate end-to-end application of the framework and surface obligations that a classification-only analysis may miss, but they do not establish inter-assessor consistency, improved compliance outcomes, or the exhaustiveness of the results produced at each stage. Thirdly, the legal interpretation of the framework remains provisional because supporting Commission guidance, harmonised standards, and national implementation of the EHDS continue to evolve.

The current paper deals with the regulatory regimes responsible for the lawful placing and continuous safe operation of medical AI systems; it does not deal with civil liability for injuries caused by those systems. With the withdrawal of the proposed AI Liability Directive in 2025, this matter is now mainly dealt with under the revised Product Liability Directive (Directive (EU) 2024/2853) (45), which classifies software and AI systems as products under a strict liability regime, along with fault-based regimes in national jurisdictions. Provider and deployer obligations defined in Stages 2 and 8 have a direct impact on civil liability, hence the interaction between the two spheres despite their being distinct.

Establishing the properties that the worked applications cannot demonstrate requires structured empirical evaluation targeting three constructs: *usability* (whether intended users can complete each stage’s artefact for a given system), *consistency* (whether independent assessors reach the same classification and produce materially equivalent artefact content), and *discriminating power* (whether the framework identifies obligations, risks, and data governance gaps that a classification-only reading does not). The proposed method is a structured expert elicitation, for example, a modified Delphi exercise, in which a multidisciplinary panel applies the framework to a shared portfolio of certified systems and iteratively converges on a predefined consensus threshold. Quantitative endpoints would include inter-rater agreement on classification decisions and artefact-field completeness (measured using Cohen’s or Fleiss’ κ, as appropriate), time to complete each artefact as a proxy for usability, and the number of distinct obligations identified relative to a classification-only baseline. Until such evaluation is undertaken, the framework should therefore be understood as a conceptually grounded governance proposal rather than a validated compliance methodology.

## 14 Conclusion

The AI Act shifts medical AI governance from point-in-time regulatory approval to continuous lifecycle assurance. Because many AI-enabled medical devices, in vitro diagnostic systems, and clinical decision-support applications fall within the high-risk category established by Article 6, that shift has immediate practical consequences for the healthcare sector. The compliance-by-design framework proposed here translates the interacting obligations of the AI Act, MDR, IVDR, GDPR, and EHDS into auditable lifecycle artefacts, giving that shift a concrete governance architecture.

The two worked examples illustrate how regulatory obligations can depend on intended purpose and deployment context rather than on the underlying AI model and how five-regime coordination can surface obligations that a single-instrument analysis may miss. Looking ahead, structured empirical evaluation through expert elicitation and Delphi-based consensus methods will be essential to establish the framework’s usability, consistency, and discriminating power across a broader portfolio of medical AI systems.

Ultimately, the success of the AI Act will depend less on the existence of legal obligations than on the ability of healthcare organisations, manufacturers, developers, deployers, and regulators to embed those obligations within routine technical, clinical, and organisational practice. The framework proposed in this paper provides one possible foundation for that transition.

## Data Availability

The original contributions presented in the study are included in the article/Supplementary Material, further inquiries can be directed to the corresponding author.
